# Simulation use in perfusion education: A 2025 survey of United States programs

**DOI:** 10.1051/ject/2025070

**Published:** 2026-06-19

**Authors:** Jared Bienstock, Bruce Searles, Edward Darling

**Affiliations:** 1 Maimonides Medical Center, Perfusion Department Brooklyn NY 11219 USA; 2 College of Health Professions, SUNY Upstate Medical University Syracuse NY 13210 USA

**Keywords:** Simulation-based education, Perfusion, Education, Simulation, Cardiopulmonary bypass

## Abstract

*Background:* Simulation-based education (SBE) is increasingly used in training students in allied healthcare, yet data on its role in United States (U.S.) perfusion programs are limited. This study surveyed perfusion programs with the purpose of assessing simulation use, curricular integration, challenges, and future directions. *Methods:* A 22-question validated survey was distributed via REDCap to all 23 perfusion program directors in the U.S. between March and April 2025. Questions addressed demographics, simulation infrastructure, curricular integration, challenges, and future directions. *Results:* The response rate was 100%. All programs use at least one simulation modality, and 82.6% use high-fidelity simulation. Most programs (69.6%) fully embed simulation into credit-bearing courses, and 73.9% grade student performance. 60.9% of programs report that they have a dedicated simulation space. 56.5% report a lack of a dedicated budget. Common challenges identified were time constraints (73.9%) and limited faculty manpower (65.2%). While all programs teach adult CPB using simulation, the incorporation of pediatric CPB, ECMO, and IABP simulation is less common. *Conclusion:* SBE is widely adopted in perfusion programs across the U.S., but standardization, faculty training, and curricular integration are necessary to optimize its impact.

## Introduction

The increased use of simulation-based education (SBE) in training healthcare students has revolutionized how students are trained and has been extensively integrated into medical education [1, 2]. At its core, it bridges classroom education with clinical practice. SBE provides a safe environment for students to practice skills and techniques, develop conceptual frameworks, hone decision-making skills, and learn from mistakes without the pressure of real-life consequences [3–5]. When compared to traditional medical training methods, SBE has been shown to improve skill acquisition and retention and allow for competency assessment [6–8]. Specifically relating to surgical and cardiothoracic residency education, SBE has been shown to improve the trainee’s basic skills and performance in the clinical environment [9, 10].

Perfusion education programs have historically augmented training with animal models and/or wet labs to teach and practice perfusion skills. With the development of high-fidelity (HF) perfusion simulators, educators began to explore what the role of this tool could have in perfusion training programs [11]. Early work has suggested that HF simulation is an effective tool for enhancing perfusion education by enabling students to practice both technical and non-technical skills. In 2011, Sistino and colleagues demonstrated that HF simulators allow students to consistently engage with realistic cardiac surgical scenarios, improving their ability to respond to emergencies, communicate effectively, and work as part of a surgical team [12]. A more recent study has validated that a HF perfusion simulation model can accurately replicate technical and physiological parameters, create a realistic and believable experience, utilize essential perfusion skills, and reliably predict real perfusion case performance [13].

The usefulness of simulation for teaching, practicing, and assessing fundamental skills is no longer debated in the profession. In the most recent revision of the Commission on Accreditation of Allied Health Education Programs, it is recommended that all perfusion schools incorporate high-fidelity simulation (HFS) into the curriculum [14]. However, specific data on the use of simulation across the aggregate of perfusion programs is unclear. Given the growth of perfusion programs in the United States (U.S.) and the lack of published data on simulation use in perfusion education, it is important to determine the current role of SBE within these programs. The purpose of this research is to assess the prevalence of SBE in U.S. perfusion education programs. This was accomplished by surveying program directors to better understand the current applications, curricular integration, challenges, and future directions of simulation across accredited perfusion schools.

## Materials and methods

### Institutional review

The State University of New York (SUNY) Upstate Institutional Review Board (IRB) has determined this project, [2100556-1], is EXEMPT from IRB review according to federal regulations.

### Survey instrument

The survey instrument consisted of 22 questions covering the following core sections: 1) Demographics, 2) Facility/Simulation Infrastructure/Equipment, 3) Curricular Integration, 4) Simulation Expenses, 5) Simulation Challenges and Barriers, and 6) Thoughts on Future Directions. The online survey tool used was the Research Electronic Data Capture application (REDCap), hosted by SUNY Upstate Medical University and developed by Vanderbilt University (Nashville, TN, USA). REDCap supports secure survey creation through the following capabilities: response deidentification, data analysis on the platform itself, exportable data packages to commonly used statistical programs, and audit trails for data manipulation and export procedure tracking [15]. The final survey instrument is publicly available via a SUNY digital repository (https://hdl.handle.net/1951/88605).

### Survey design and validation:

Initially, our research group developed and refined questions surrounding the use of simulation in perfusion education programs. To ensure the survey questions were free from bias and ambiguity, validity testing was performed for each question. First, volunteer subject matter experts (SMEs) were recruited to provide face and content validity to ensure question clarity, relevance, and to ensure that the question set adequately covered the topic. From the SME feedback, several refinements were made to the survey. Next, survey construct validation of wording, sentence structure, and significance was performed using the University of Memphis’ Question Understanding Aid (QUAID) Tool (Memphis, TN, USA) [16]. Next, a REDCap expert was directed to review the survey format, structure, branching logic, and flow to ensure that no technical errors existed in each question’s design. Finally, to confirm that the questions were clear to the target participants, a survey pilot was conducted with five program directors representing diverse program types, including certificate and master’s programs as well as established and newer programs.

### Target population, time frame, and recruitment method:

The target population included the directors of 22 accredited perfusion programs, as well as the director of one newly established program currently under accreditation review in the U.S. (*n* = 23). A list of twenty-three perfusion programs and their director contact information was obtained from the Perfusion Program Directors Council website [17]. Survey responses were collected through voluntary survey completion between March and April of 2025. Using the REDCap collection feature, a standardized email was sent to each program director containing the survey description and unique survey link. For those who did not initially respond, a follow-up email reminder was set to repeat every week for 5 weeks.

### Data presentation

Survey data is expressed as a percentage (%) of total responses.

## Results

All twenty-three program directors completed the survey, providing a 100% response rate. Of the twenty-three programs, 13 (56.5%) have been operational for greater than 11 years, 2 (8.7%) for 6–10 years, and 8 (34.8%) for 5 or fewer years. The primary degrees conferred by the programs are master’s degree 17 (74%) and post-bachelor certificate 6 (26%). Median program duration is 21 months (average = 19.87 ± 3.1 months) with a range of 12–24 months. The incoming 2025 student class size for programs ranged from 0 to 44 students (median = 12 students/program, mean = 14.04 ± 9.23 students/program). Across all programs, the total number of 2025 incoming students is 323.

Of the twenty-three programs, the mean core perfusion faculty is 4.74 ± 2.73 persons (median = 4, range 1–10 persons. Of these core perfusion program faculty members, those who teach/conduct simulation are 3.26 ± 2.18 persons (median = 3, range 1–10 persons). In ten programs (43%), all core perfusion faculty teach/conduct simulation, while not every faculty member will teach/conduct simulation in the remaining 13 programs (57%).

The reported forms of simulation programs utilized in the curriculum are shown in Table 1. All programs use at least one mode of simulation, and the application of HFS for education and training was reported by over 80% of the program directors.

**Table 1:** Forms of simulation used in program curriculum.

Forms of simulation	Program no (%)
High-fidelity simulation	19/23 (82.6%)
Low-fidelity/wet labs	17/23 (73.9%)
Video/computer simulation	10/23 (43.5%)
Animal extracorporeal labs	2/23 (8.7%)

The infrastructure in which perfusion simulation is conducted is shown in Table 2. Briefly, 14 programs (60.9%) reported that they have a dedicated simulation space/mock O.R. available to teach/conduct simulation. Two of the 4 programs that selected “Other” had no campus facility and either rented simulation space or used the O.R. for simulation when available. The majority of perfusion programs (56.5%) do not have a dedicated budget for simulation expenses. The use of student lab fees to offset simulation expenses was reported by 9 (39.1%) program directors.

**Table 2:** Physical facility for the conduct/teaching of perfusion simulation.

Location of simulation facility	Program no (%)
Perfusion programs dedicated simulation room(s)/Mock O.R.	14/23 (60.9%)
Perfusion programs dedicated multi-purpose space	8/23 (34.8%)
Institutional campus shared simulation center	6/23 (26.1%)
Shared multipurpose space	6/23 (26.1%)
Other	4/23 (17.4%)

Most students have access to simulation practice both in class and for independent practice outside of class (Table 3). Ten programs reported not providing students with 24-hour access to independent simulation practice outside of class-scheduled sessions.

**Table 3:** Student access to simulation practice.

Simulation practice access times	Program no (%)
During specific course times (faculty-led)	16/23 (69.6%)
Outside of class schedule for independent practice (open to students 24 h)	13/23 (56.5%)
Outside of class schedule for independent practice (business hours)	8/23 (34.8%)

**Figure 1:**
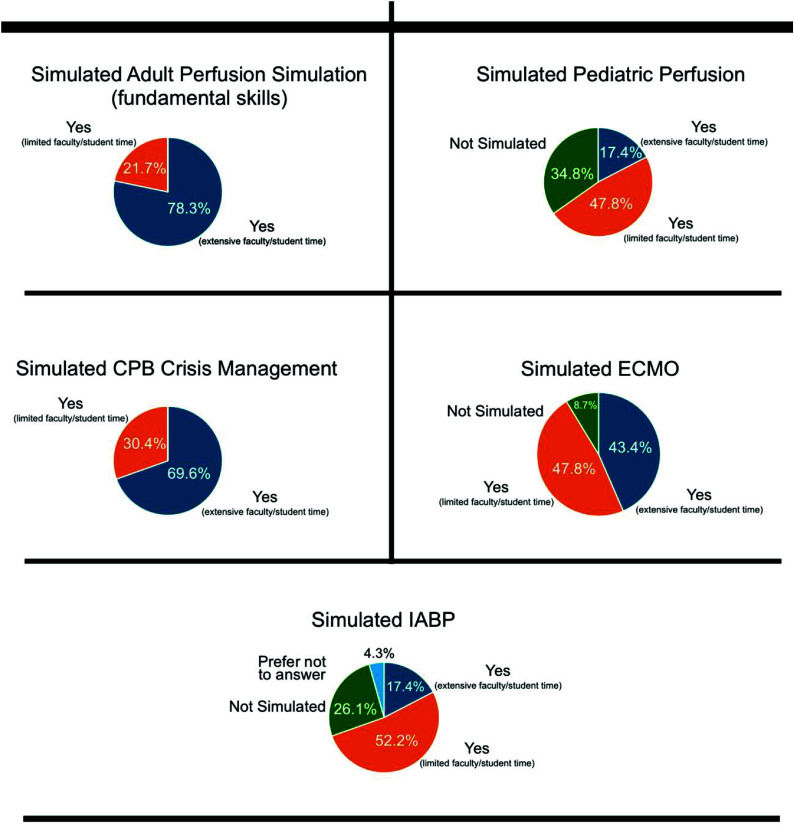
The application of perfusion simulation across the categories of Adult CPB, Pediatric CPB, Crisis Management, ECMO, and IABP.

Program directors applied simulation to demonstrate scenarios in the following categories: adult cardiopulmonary bypass (CPB), pediatric CPB, crisis management, extracorporeal membrane oxygenation, and intra-aortic balloon pump (IABP) reported by program directors are shown in Figure 1.

All programs simulate adult CPB fundamental skills, with 18 programs (78.3%) describing this with extensive faculty/student commitment. Four programs (17.4%) described their pediatric CPB simulation as extensive, while 11 (47.8%) reported limited pediatric simulation. Eight programs (34.8%) did not do any pediatric CPB simulation. All programs do some degree of simulated crisis management exercises with their students, 16 (69.6%) extensive and 7 (30.4%) limited.

While nearly all programs connect and integrate simulation to the curriculum, the extent of that incorporation is variable (see Table 4).

**Table 4:** Perfusion programs curricular integration and assessment of simulation performance.

Curricular integration of simulation	Program no (%)
Fully embedded into credit-bearing simulation courses	16/23 (69.6%)
Augmenting teaching in credit-bearing courses	8/23 (34.8%)
Used for general demonstrations	8/23 (34.8%)
Not integrated into the curriculum	1/23 (4.3%)
Simulation activities are part of coursework and contribute to students’ grades	17/23 (73.9%)
Simulation activities are part of coursework for competency assessment without grading	5/23 (21.7%)
Simulation activities are not part of coursework and are for practice or demonstration purposes only	3/23 (13%)

Most programs (52.2%) do not record simulation sessions for students to review their performance, while 39.1% do, or sometimes do, record sessions. Two programs preferred not to answer this question. All programs report debriefing following simulation activities related to coursework, although 4 programs (17.4%) would describe debriefing as minimal.

Six programs (26.1%) reported that perfusion simulation is used at their institutions in interprofessional education (IPE) (perfusion students training with other allied health students).

There are several challenges that program directors have encountered when using SBE in their programs. The most frequently reported challenges are time constraints (73.9%) and the lack of faculty manpower (65.2%). Two other challenges listed by programs included “keeping all the technology working is a big-time commitment” and “support from the simulation company when items break.” None of the programs reported experiencing challenges related to a lack of student buy-in. A complete list of challenges is shown in Table 5.

**Table 5:** Challenges encountered by programs when using simulation-based education.

Challenges to simulation-based education	Program no (%)
Time constraints	17/23 (73.9%)
Lack of faculty manpower	15/23 (65.2%)
Difficulty procuring perfusion hardware and disposables	12/23 (52.2%
Lack of space and infrastructure	10/23 (43.5%)
Limited access to simulation equipment	8/23 (34.8%)
Lack of funding	7/23 (30.4%)
Difficulty integrating simulation into the curriculum	3/23 (13%)
Resistance from faculty	3/23 (13%)
Other	2/23 (8.7%)
None	1/23 (4.3%)

With regard to integrating simulation in coursework, 15 (65.2%) program directors responded that they plan on increasing the integration of simulation into coursework, while the remaining 8 (34.8%) programs indicated that the level of simulation integration will not change. None of the programs reports a plan to decrease simulation curricular integration.

Additionally, program directors were asked how important they feel simulation will be in the future of perfusion education. Eighteen of the program directors (78.3%) responded with very important or important, while 5 (21.7%) responded with somewhat important or not very important, and zero participants indicated that it was not important at all. When queried on their beliefs surrounding whether various emerging simulation technologies and trends should be incorporated into perfusion education. of the twenty-three program directors, 12 (52.2%) selected that virtual reality (VR) should be incorporated into perfusion simulation education, while 6 (26%) program directors preferred not to answer or did not believe that these emerging simulation technologies should be incorporated into perfusion education. One respondent believed that these technology tools may provide an opportunity to supplement, but did not believe that they will/should/can replace HFS. These results are demonstrated in Table 6.

**Table 6:** Emerging technologies and trends in perfusion simulation.

Select the emerging technologies or trends that you believe should be incorporated into perfusion simulation education	Selected
Virtual reality (VR) simulations	12/23 (52.2%)
Artificial intelligence (AI) driven simulations	11/23 (47.8%)
Gamification elements in simulation	10/23 (43.5%)
Augmented reality (AR) simulations	9/23 (39.1%)
Prefer not to answer	3/23 (13.0%)
I do not believe these should be incorporated	3/23 (13.0%)
Other	1/23 (4.3%)

When program directors were asked if they would have any recommendations for improvements and/or changes as it relates to SBE in perfusion programs, their selected answers are shown in Table 7. Briefly, the top 2 selected recommendations are increased simulation hours in curriculum 11 (47.8%) and more faculty training on simulation techniques 11 (47.8%). Three program directors did not have any recommendations, and 2 others preferred not to answer. There were 2 program directors who offered other write-in recommendations, which were (i) “Wider adoption by schools – the technology we have is not the limitation – national application is the rate-limiting step” and (ii) “Higher accreditation standards.”

**Table 7:** Program directors recommendations.

Would you recommend any improvements/changes regarding the use of simulation-based education in perfusion programs?	Selected
Increased simulation hours in the curriculum	11/23 (47.8%)
Faculty training on simulation techniques	11/23 (47.8%)
More standardized simulation scenarios	8/23 (34.8%)
Better access to state-of-the-art simulation equipment	8/23 (34.8%)
No recommendations	3/23 (13.0%)
Other	2/23 (8.7%)
Prefer not to answer	2/23 (8.7%)

The survey provided an option for program directors to add any additional comments. There were six respondents, and these open-ended comments are shown in Table 8.

**Table 8:** Open-ended additional comments.

Additional comments
The ease of use for the simulation equipment is a large learning curve from year to year. Annual training is needed to maintain the quality of simulations.
We are a new program, yet to start our first cohort, so I chose not to answer some questions based on NO experience with our students/simulation. I answered based on our anticipated SIM experience with students.
Sims are limited by equipment and sim faculty education. In general, the simulators need to get more user-friendly.
Thank you for this survey, helpful for our program to petition sponsoring organizations for resources to develop a simulation program for the school.
[At our center] there is no shortage of Actual Reality cases for training. The greatest value for simulation in this environment would be to train for emergencies, yet it is difficult to truly simulate emergencies. [For a high-volume education program], it is difficult to justify costs to simulate reality

## Discussion

The collective use of simulation within the field of perfusion education in the U.S. has not been previously surveyed. This underscores the importance of identifying baseline usage patterns and trends among schools to determine the future direction of SBE to ultimately improve student training and potentially standardize the approach of simulation integration within programs. Prior research in perfusion education and simulation also reinforces the need to assess SBE use in perfusion programs across the U.S. Over the last decade, there has been notable growth in both the number of accredited perfusion programs and the adoption of simulation technologies. As of 2025, there are twenty-three perfusion programs in the U.S., up from about 16 years ago.

In 2010, the American Society of Extracorporeal Technology and the American Academy of Cardiovascular Perfusion jointly surveyed the perfusion community on a variety of topics, including the role of simulation within perfusion schools. Their findings showed that, out of 427 respondents, 17.10% (*n* = 73) reported that their “perfusion school used simulation as a peripheral adjunct” to clinical training, while only 6.79% (*n* = 29) reported that simulation was a “a major curricular component of” their clinical training [18]. A 2020 article published by the American Academy of Cardiovascular Perfusion also described simulation use in perfusion education as “underutilized” compared to other health professions [19].

A key finding of this 2025 survey is that all programs incorporate at least one simulation modality (i.e., low fidelity/wet labs, video/computer simulation, or animal labs). The use of HFS for education and training was reported by more than 80% of the program directors. 78.3% of the programs teach adult CPB through simulation, describing it with extensive faculty/student commitment. Other core topics, such as pediatric perfusion, crisis management, ECMO, and IABP are also taught in simulation but to lesser degrees. These findings suggest that while early adoption may have been limited, simulation is now a core component of modern perfusion education.

## Infrastructure and resources

The authors identified that there is variation in simulation infrastructure and student access to practice time. While 60.9% of perfusion programs have a dedicated simulation room(s), other programs rely on shared or even rented simulation spaces. Limited access to physical simulation environments could hinder the frequency and consistency of SBE use. Programs with access to dedicated simulation spaces may be better positioned to conduct comprehensive simulated scenarios, integrate IPE, and provide students with time for independent practice. On the other hand, when simulation settings are shared, it can pose scheduling conflicts or prevent students from having opportunities outside of class to practice. Over half (56.5%) of the programs report that students have 24-hour access for independent practice. Limited access may impact students’ abilities to develop confidence, knowledge, skillsets, and competence due to having limited access to simulation resources.

## Curricular integration and assessment

This survey demonstrates that curricular integration of simulation differs widely by clinical content, on which topics are being taught, and to what extent. According to Elendu and colleagues, “Integrating Simulation into existing curricula requires careful planning and coordination to ensure it complements traditional training methods and enhances overall learning outcomes” [2]. It is essential for faculty to be involved in curriculum development to make sure that simulation activities align with the established learning objectives [2]. While all programs simulate adult CPB, simulation use in pediatric perfusion is less consistent. Only 17.4% of programs reported extensive pediatric simulation, and 34.8% do not conduct any pediatric simulation at all. This gap between adult and pediatric simulation practices may be due to a number of factors. The stated time constraints surrounding simulation may have deprioritized pediatric simulation in favor of the more common adult CPB cases. Perhaps future curricular planning should increase the incorporation of pediatric simulation training scenarios into education. This may help improve student preparedness and competency in a highly technical domain.

Simulation use in ECMO and IABP was assessed in this survey and revealed areas for potential curricular growth. Simulation use in ECMO education was reported by 47.8% of the programs that taught limitedly. Similarly, 52.2% of programs report that IABP simulation education is covered to a limited extent, and notably, 26.1% do not use simulation to teach IABP skills. Given the prominent role that perfusionists play in ECMO and IABP management, expanding SBE in these areas could enhance student preparedness and skill levels. Although these modalities are typically considered ancillary procedures within perfusion practice, their complexity reinforces the need for greater simulation training in these areas. The limited curricular integration of simulation into these domains is of concern, considering that critical decision-making is required with these domains and that these modalities are often initiated in emergent environments. With that being said, all programs report utilizing simulation for crisis management training, although to different degrees. Most programs dedicate extensive time to crisis management simulation (69.6%), whereas 30.4% simulate crisis management but with limited faculty and student time.

Only 8.7% of the programs currently use animal labs in their training. Historically, animal labs were a key part of perfusion education, providing hands-on experience with live animals and thus live physiological responses. However, the use of animal labs has largely disappeared, according to survey results. This may be due to ethical concerns around animal welfare and regulatory restrictions, which make animals less accessible. Animals may also be inaccessible due to the costs of procurement, the need for dedicated lab space, and the need for veterinarians to cannulate and perform anesthesia. Additionally, advances in simulation now offer highly realistic environments with simulated patients that mimic human physiology without the ethical, logistical, and financial considerations associated with using live animals. This shift reflects evolving educational standards, which offer modernized training tools to prepare students without the use of animals.

One of the more prominent findings from the survey is that nearly all the programs (69.6%) fully embed simulation into their curricula with credit-bearing courses. 73.9% of the programs report assigning grades based on simulation performance. Only one program reported not integrating simulation into the curriculum. These results suggest that simulation is not only perceived as a supplemental activity, but it is seen as an important educational tool for competency-based learning and assessment. Furthermore, there is also diversity in how simulation is assessed. 21.7% of programs use simulation for competency assessment without grading, and 13% report that simulation activities are not part of coursework and are for practice or demonstration only. Programs that assess simulation performance with grades provide emphasis on measurable outcomes. Grading performance in simulation provides a metric for students to understand where they are in their learning experience. At the same time, it also provides faculty with quantitative data to help students improve. Expanding grading into the curriculum of all programs could help establish program consistency in how simulation is integrated and assessed. Survey results indicate that the majority of program directors (65.2%) are planning to expand simulation use within their curriculum, and none of the programs report a plan to decrease simulation curricular integration.

## Future direction and challenges

The majority of program directors (78.3%) feel that simulation will play an important or very important role in perfusion education. About half of program directors (52.2%) favor utilizing VR, while less than half of program directors support the use of augmented reality, artificial intelligence-simulations, and gamification. Few program directors (13%) felt that emerging technologies should not be incorporated into perfusion simulation. These results suggest that there may be uncertainty in using these emerging technologies. This may be because technology evolves so quickly, and faculty may not yet be familiar with how it works or why it is beneficial. Programs may also not use these due to costs and a lack of validated perfusion-specific tools. Additionally, time constraints (73.9%), lack of faculty manpower (65.2%), and limited funding (30.4%) could contribute to a lack of interest. One program director also noted that emerging technologies should supplement, not replace, HFS. Although the majority of programs may not express interest in these technologies, they can help improve learning retention and performance.

Despite the overwhelming consensus in medical literature on the importance of simulation use in the future of perfusion education, 21.7% responded with believing that simulation is somewhat or not very important in the future. This may exist due to obstacles and challenges that these program directors experience in implementing meaningful simulation. The results offer insight into a range of underlying challenges that impact the future of simulation. These barriers include, but are not limited to, issues with time constraints (73.9%), difficulty procuring equipment (52.2%), lack of space (43.5%), and lack of funding (30.4%), to name a few. A few program directors report difficulty with implementation due to resistance from faculty (13%). In regard to lack of funding, the survey found that 39.1% of program directors report the use of student lab fees to offset simulation expenses as a solution/supplement to the budget. According to Elendu and colleagues, one of the most predominant challenges with simulation technologies is their high cost and that it can be resource intensive [2]. HFS “programs require substantial financial investment” [2]. Purchasing equipment can be unaffordable for many educational institutions, particularly those with limited funding. According to Davis and colleagues, the “inability to articulate return on investment to administrators may create a snowball effect leading to lack of buy-in and inadequate resource allocation, making it even more difficult to launch or maintain a simulation program” [6].

Faculty development is critical in all aspects of simulation. Lack of faculty manpower (65.2%) was also reported as a challenge in implementing SBE. It is important that facilitators, or dedicated simulation faculty, acquire knowledge and skills necessary to educate at a high degree [6]. Successful use of SBE depends on the proficiency of the faculty in “both the technical aspects…and the pedagogical skills necessary to facilitate learning” [2]. Simulation educational skillsets and knowledge can be acquired via professional development opportunities, conferences, and training workshops. Educational institutions can/should invest in professional development for faculty to stay current in simulation education techniques and skills.

There is a valid perspective among clinicians who feel confident in training primarily on real patients, since that was their own experience, and correspondingly, some high-volume training programs may believe that in vivo learning is best suited for teaching and view simulation as primarily valuable for training emergencies. However, this raises an important question: should new skills be learned directly on real patients? [20] There may be significant value in shifting the learning curve of the student into the simulation lab prior to patient encounters. In fact, in a large survey of the perfusion community, nearly 83% of practicing perfusionists indicated perfusion schools should document students’ competency in specific pre-clinical skills before allowing students to practice these skills on live patients [18].

Notably, none of the programs reported challenges with obtaining student buy-in. This suggests that students generally recognize the value of SBE and are engaging in this learning process. Programs may therefore be able to focus on other challenges, such as faculty training or curriculum integration, without having to address student resistance.

## Recommendations from program directors

Nearly half of the survey respondents recommend increasing simulation hours and enhancing faculty training. This highlights the need for further, clearly defined establishment of national standards of SBE use in perfusion education and curriculum. Faculty simulation training programs, standardization of simulation scenarios, and a shared simulation repository to standardize these scenarios may assist perfusion programs in implementing more vigorous SBE practices. This is supported by the results of this survey, where two program directors offered other write-in recommendations, which were (i) “wider adoption by schools, the technology we have is not the limitation, and national application is the rate-limiting step” and (ii) “Higher accreditation standards.”

Future research and pilot programs focusing on simulation curriculum integration and the use of emerging technologies are needed to help programs implement simulation in ways that improve student training.

## Limitations

There were several limitations to this study. One limitation is that all responses were made by program directors, which may have resulted in response bias. It is possible that some programs overestimated their simulation integration, while others may have underreported challenges. This could be a result because the use of terms such as limited and extensive, regarding time commitments to utilizing simulation to teach, was not clearly defined, and left to interpretation. Another limitation is that this study solely focused on frequencies and integration, and not on educational outcomes. Although the response rate was 100%, it is important to note that this study exclusively surveyed programs within the U.S. and excluded international programs.

## Conclusion

With the data collected and reported in this survey, perfusion programs may gain a better understanding of how simulation technologies are currently being integrated into perfusion education across the U.S. Faculty and program directors can use these findings to evaluate their own curricular structures, degree of curricular integration, identify opportunities for change, and advocate to their administration for increased funding for simulation resources and activities. The survey also helps programs recognize underrepresented areas in training, such as pediatric perfusion, ECMO, and IABP, suggesting the need for developing simulation content in these domains. SBE is widely adopted in perfusion programs across the U.S., but standardization, faculty training, and curricular integration are necessary to optimize its impact.

## Data Availability

All available data is incorporated into the article.
